# Co-expression of nitrogenase proteins in cotton (*Gossypium hirsutum* L.)

**DOI:** 10.1371/journal.pone.0290556

**Published:** 2023-08-24

**Authors:** Yimin Shang, Wenfang Guo, Xiaomeng Liu, Lei Ma, Dehu Liu, Sanfeng Chen

**Affiliations:** 1 College of Biological Sciences, China Agricultural University, Beijing, China; 2 Biotechnology Research Institute, Chinese Academy of Agricultural Sciences, Beijing, China; ICAR-National Rice Research Institute, INDIA

## Abstract

Chemical nitrogen fertilizer can maintain crop productivity, but overuse of chemical nitrogen fertilizers leads to economic costs and environmental pollution. One approach to reduce use of nitrogen fertilizers is to transfer nitrogenase biosynthetic pathway to non-legume plants. Fe protein encoded by *nifH* and MoFe protein encoded by *nifD* and *nifK* are two structural components of nitrogenase. NifB encoded by *nifB* is a critical maturase that catalyzes the first committed step in the biosynthesis of nitrogenase FeMo-cofactor that binds and reduces N_2_. Expression of the *nifB*, *nifH*, *nifD* and *nifK* is essential to generate plants that are able to fix atmospheric N_2._ In this study, the four genes (*nifB*, *nifH*, *nifD* and *nifK*) from *Paenibacillu polymyxa*WLY78 were assembled in plant expression vector pCAMBIA1301 via Cre/LoxP recombination system, yielding the recombinant expression vector pCAMBIA1301-*nifBHDK*. Then, the four *nif* genes carried in the expression vector were co-introduced into upland cotton R15 using *Agrobacterium tumefaciens*-mediated transformation. Homozygous transgenic cotton lines B2, B5 and B17 of T_3_ generation were selected by PCR and RT-PCR. qRT-PCR showed that *nifB*, *nifH*, *nifD* and *nifK* were co-expressed in the transgenic cottons at similar levels. Western blotting analysis demonstrated that NifB, NifH, NifD and NifK were co-produced in the transgenic cottons. Co-expression of the four critical Nif proteins (NifB, NifH, NifD and NifK) in cottons represents an important step in engineering nitrogenase biosynthetic pathway to non-legume plants.

## Introduction

Nitrogen (N) fertilizer can maintain crop productivity, but overuse of chemical N fertilizers leads to economic costs and environmental pollution [1]. One approach to reduce use of N fertilizers is to transfer biological nitrogen fixation to non-legume crops that can fix nitrogen [2, 3].

Biological nitrogen fixation, conversion of N_2_ to NH_3_, is catalyzed by nitrogenase enzyme that is distributed in a few bacteria and archaea. Nitrogenase is composed of two component proteins, MoFe protein (also termed as NifDK protein) and Fe protein (also termed as NifH protein). Fe protein is a homodimer bridged by an intersubunit (4Fe-4S) cluster that serves as the obligate electron donor to MoFe protein. MoFe protein is a heterotetramer that contains two metalloclusters: P cluster (8Fe−7S) and FeMo-cofactor (Mo−7Fe−9S−C−homocitrate) which binds and reduces N_2_ [4, 5]. Studies on N_2_-fixing models *Azotobacter vinelandii* and *Klebsiella oxytoca* have revealed that nitrogenase synthesis and maturation require at least 16 genes. In addition to *nifH*, *nifD* and *nifK* encoding structural subunits of nitrogenase, *nifB*, *nifE nifN*, *nif*X, *nifQ*, *nifV*, *nifW*, *nifZ*, *nifM*, *nifU*, *nifS*, *nifF* and *nifJ* contribute to synthesis of FeMo-cofactor, P cluster and 4Fe-4S cluster and electron transport [6–8]. Our study has revealed that a minimal *nif* gene cluster composed of 9 genes (*nifB nifH nifD nifK nifE nifN nifX hesA nifV*) from *Paenibacillus polymyxa* WLY78 enabled *Escherichia coli* to fix nitrogen, and deletion analysis has revealed that the six genes *(nifB*, *nifH*, *nifiD*, *nifK*, *nifE* and *nifN*) are essential for nitrogenase activity [9]. FeMo-cofactor is synthesized independently and then inserted into apo-NifDK. NifB is a radical S-adenosyl methionine (SAM) enzyme that catalyzes the formation of NifB-co, a [8Fe-9S-C] cluster which is a precursor for syntheses of FeMo-cofactor of Mo-nitrogenase [10–13]. NifB proteins have great diversity in their protein architectures among bacteria and archaea [14–16].

There has been a long-standing interest in engineering nitrogenase biosynthetic pathway in non-legume crops that can fix nitrogen [2, 3, 17, 18]. Active nitrogenase Fe protein was detected in tobacco when *nifH* and *nifM* from *A*. *vinelandii* were co-expressed in tobacco chloroplast [19]. Sixteen *nif* gengs (*nifB*, *nifD*, *nifE*, *nifF*, *nifH*, *nifJ*, *nifK*, *nifM*, *nifN*, *nifQ*, *nifS*, *nifU*, *nifV*, *nifX*, *nifY* and *nif*Z) from *K*. *oxytoca* were individually expressed using a transient expression system and these Nif proteins were individually targeted to the mitochondrial matrix [20]. NifB-co, a precursor of FeMo-cofactor, was produced in transgenic rice when NifB from the archaea *Methanocaldococcus infernus* and FdxN from *A*. *vinelandii* were co-expressed [21]. Functional *nifB* was produced in tobacco chloroplasts and mitochondria when *nifB* from *Methanosarcina acetivorans* or *M*. *infernus* and NifS, NifU and FdxN from *A*. *vinelandii* were co-expressed [22]. Although engineering nitrogen fixation genes in non-legume plants shows an attractive prospect, it is still a great challenge to achieve the simultaneous expression of multiple genes in plants.

In this study, we use Cre/LoxP recombination system to assemble four genes (*nifB*, *nifH*, *nifD* and *nifK*) from *P*. *polymyxa*WLY78 in expression vector pCAMBIA1301. Then, the four *nif* genes carried in the expression vector ware co-introduced into upland cotton R15 mediated by *Agrobacterium tumefaciens*. Homozygous transgenic lines B2, B5 and B17 of T_3_ generation were identified by PCR and RT-PCR. qRT-PCR and western blotting analysis showed that *nifB*, *nifH*, *nifD* and *nifK* were co-expressed in transgenic cottons. The Cre/LoxP recombination system provides one efficient strategy for co-expression of multicomponent nitrogenase, and the stable expression of nitrogenase subunits in homozygous transgenic cottons demonstrates that feasibility of reconstituting nitrogen fixation pathway in non-legumes.

## Materials and methods

### Plant materials and bacterial strains

Upland cotton (*Gossypium hirsutum* L.) R15 was used for introduction of *nif* genes. *Escherichia coli* SW106 was used as the host for DNA assembly to plant expression vector, and *E*. *coli* DH5a was employed for routine gene cloning. *Agrobacterium tumefaciens* strain LBA4404 was used for plant transformation. When necessary, kanamycin and ampicillin were used at 50 mg/L. The coding regions of the 4 genes (*nifB nifH nifD nifK*) from *P*. *polymyxa* WLY78 were synthesized according to the codon bias in the *S*. *cerevisiae* genome by GenScript Co., Ltd. (Nanjing, China) (S1 Table).

### Construction of the recombinant plant expression vector

Plant expression vector used in this study is pCAMBIA1301 that was modified by adding LoxP sequences as described by Ma et al [23]. Three satellite vectors pOSB103, pOSB104 and pOSB202 were used (Table 1) [23]. The codon-optimized coding sequences of *nif* genes (*nifB*, *nifH*, *nifD* and *nifK*) were PCR amplified with primers (S2 Table).

**Table 1:** Recombinant satellite plasmids carrying *nif* genes, promoters and terminators.

Gene	promoter	terminator	Resistance	Restriction site at 5’ end	Restriction site at 3’ end	Satellite vector	Recombinant satellite plasmid
*nifB*	nos	nos	Amp^r^	*Xho* I	*Bsr*G I	pOSB103	pOSB103-*nifB*
*nifH*	35S	35S	Amp^r^	*Xho* I	*Bsr*G I	pOSB202	pOSB202-*nifH*
*nifD*	ocs	ocs	Amp^r^	*Xho* I	*Bsr*G I	pOSB104	pOSB104-*nifD*
*nifK*	35S	35S	Amp^r^	*Xho* I	*Bsr*G I	pOSB202	pOSB202-*nifK*

### Multiple rounds of recombination reactions

For Cre/loxP-mediated plasmid co-integration, the recombinant satellite plasmid pOSB and expression vector pCAMBIA1301 were co-electroporated into cells of *E*. *coli* strain SW106, in which the *cre* gene was induced by adding arabinose [23]. A positive transformant of *E*. *coli* strain SW106 was screened on LB plates containing Kanamycin and Ampicillin. The resulting plasmid was isolated from the positive transformant and then it was introduced into *E*. *coli* DH5a. The resulting plasmid was digested with appropriate restriction endonuclease to produce linear plasmid and then the linear plasmid was ligated into circle plasmids by using T4 ligase. Then, another Cre/loxP-mediated plasmid co-integration was performed. Finally, a positive transformant of *E*. *coli* DH5a carrying the recombinant expression vector pCAMBIA1301-*nifBHDK* with four *nif* genes (*nifB*, *nifH*, *nifD* and *nifK*) was obtained on LB plates containing Kanamycin.

### Genetic transformation of cotton and selection of transgenic plants

Expression vector pCAMBIA1301-*nifBHDK* carrying hygromycin resistance gene (Hyg) was transformed into *A*. *tumefaciens* LBA4404. Hypocotyl explants from cotton seedlings were transformed using previously described methods [24, 25]. Cotton hypocotyls and *A*. *tumefaciens* carrying pCAMBIA1301-*nifBHDK* were co-cultured. The hypocotyls were transferred to a callus medium to induce the formation of embryoid bodies and then further cultured to form transgenic cotton seedlings. The whole genetic transformation process was conducted in a 28°C incubator (16 h light/8 h dark). The transgenic seedlings were subsequently grafted on cotton rootstocks (25-day seedlings of island cotton) to obtain T_0_ transgenic cotton plants. T_0_ transgenic cotton plants with Hyg were identified by PCR and RT-PCR. T_0_ transgenic cotton plants containing the target genes (*nifBHDK*) were continued to be grown under culture conditions to facilitate selfng and boll formation. Seeds of T_0_ transgenic cotton plants were collected from each plant and grown under greenhouse conditions to self and form bolls. T_1_ generation seeds were collected from each transgenic cotton plant and then planted in fields, and then T_2_ transgenic cotton plants were obtained.

### PCR, RT-PCR and qRT-PCR

PCR was used to assay transgenic cotton plants. Genomic DNA was isolated from fresh leaves of cotton plants (transgenic cotton and non-transgenic cotton) using the CTAB method [26]. The target genes (*nifB*, *nifH*, *nifD* and *nifK*) were identified by PCR with primers nifB-F, nifB-R, nifH-F, nifH-R, nifD-F, nifD, nifK-F and nifK-R (S3 Table). The genomic DNA of non-transgenic cotton leaves was used as the negative control, and the recombinant vector pCAMBIA1301-*nifBHDK* containing the tetravalent *nif* genes was used as the template of the positive control.

For RT-PCR and qRT-PCR, total RNA was isolated from Leaves of 30-day cotton seedlings, including the transgenic cotton plants and non-transgenic cotton plants, by using a total RNA Extraction and Purifcation Kit (Sangon Biotech, Shanghai, Co., Ltd. China). Reverse transcription synthesis of cDNA was performed using RevertAid Premium Reverse Transcriptase (Thermo ScientifcTM EP0733). RT-PCR and qRT-PCR were performed with cDNA as template. Primers for RT-PCR were shown in S3 Table and primers for qRT-PCR were listed in S4 Table. The *GhUBQ7* in cotton plants was used as a control. T_1_ generation seeds were collected from each transgenic cotton plant containing the target genes (*nifBHDK*) and the process was repeated for 2–3 generations until homozygous transgenic cotton lines were obtained.

### Western blot analysis of Nif protein expression

For extraction of total protein, 0.1g of young leaves (30-day cotton seedlings) was put into a small tube, and then100 μL of PBST buffer (1000 mL containing 8 g NaCl, 0.2 g KCl, 3.63 g Na_2_HPO_4_·12H_2_O, 0.24 g KH_2_PO_4_ and 0.05% Tween-20, pH 7.4) and a few glass beads were added to the tube. The tube was violently shaken on the tissue homogenizer to break cotton leave tissues and then was centrifuged at 12000 g for 5 min. Protein samples (30 μL) was mixed with 30μL of 2×SDS gel-loading buffer and boiled for 10 min, and then, 20 μL was loaded onto 12% separating gels and 5% stacking gel for SDS-polyacrylamide gel electrophoresis (SDS-PAGE). The Nif proteins were detected using the 4 antibodies (anti-NifB, anti-NifH, anti-NifD and anti-NifK) that were raised against the proteins NifB, NifH, NifD and NifK of *P*. *polymyx*a WLY78 that were expressed and purified from *E*. *coli* BL21. Western blotting was done by using Western Blot Kit (CoWin Biosciences, China).

## Results

### Construction of plant multigene expression vector

The codon-optimized sequences of four *nif* genes (*nifB*, *nifH*, *nifD* and *nifK*) were shown in S1 Table. Plant expression vector used in this study is pCAMBIA1301 that was modified by adding LoxP sequences (ATAACTTCGTATAGCATACATTATACGAAGTTAT) as described by Ma et al [23]. Three satellite vectors pOSB103, pOSB104 and pOSB202 carried nos (nopaline synthase gene) promoter/nos terminator, ocs (octopus alkali synthase) promoter/ocs terminator and 35S (Caulifower mosaic virus 35S, CaMV) promoter/35S terminator, respectively (Table 1).

The codon-optimized coding sequences of *nif* genes (*nifB*, *nifH*, *nifD* and *nifK*) were PCR amplified with primers (S2 Table). Each of the 4 *nif* genes (*nifB*, *nifH*, *nifD* and *nifK*) was correspondingly ligated to *Xho*I and *Bsr*GI digested satellite vectors (pOSB103, pOSB202, pOSB104 and pOSB202), generating 4 recombinant satellite plasmids (pOSB103-*nifB*, pOSB202-*nifH*, pOSB104-*nifD* and pOSB202-*nifK*) (Table 1). These recombinant satellite plasmids were verified by sequencing.

**Fig 1:**
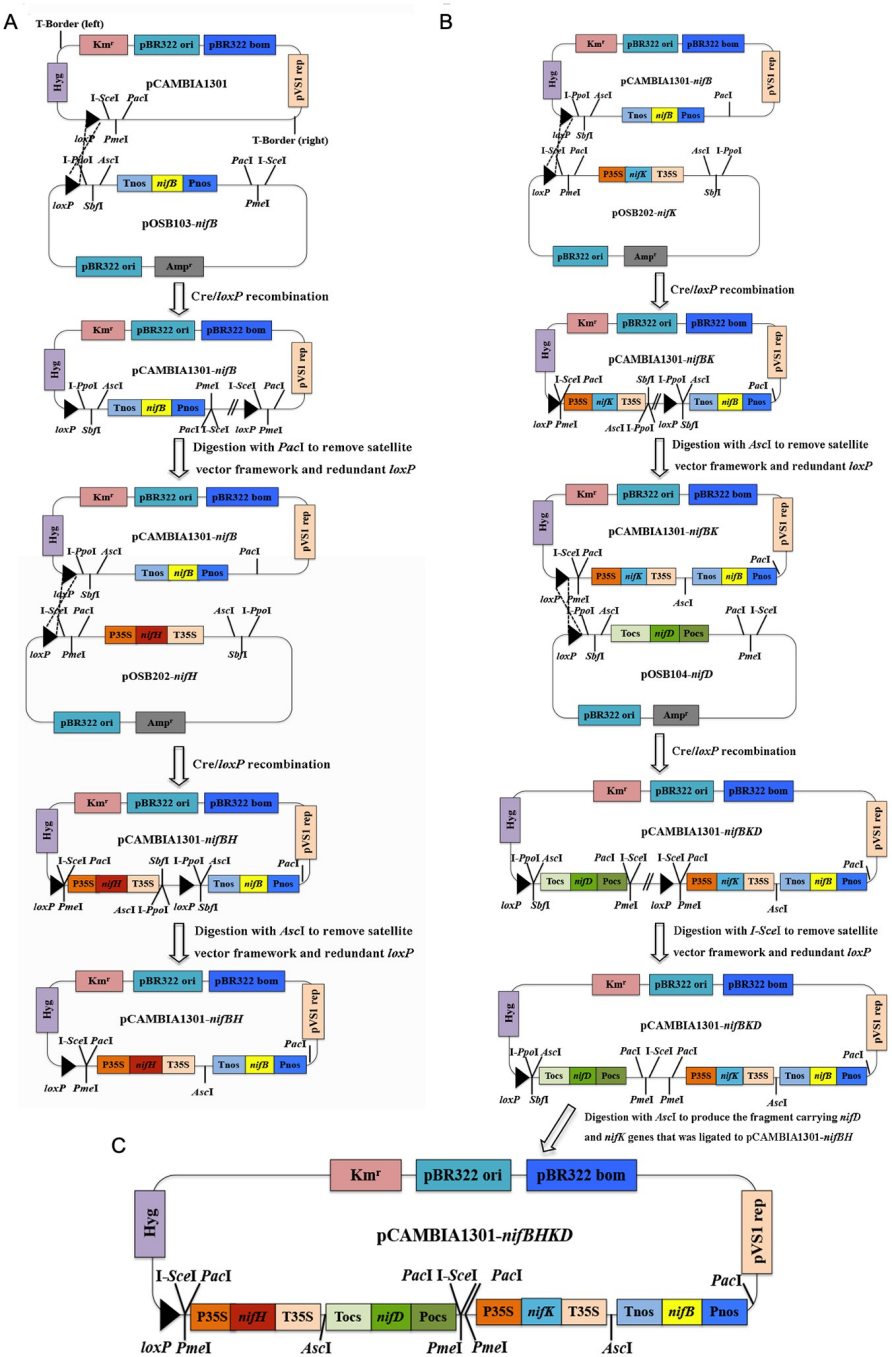
Schematic diagram for the process of *nif* genes stacking to plant expression vector one by one via Cre/LoxP recombination. (A) *nifB* and *nifH* stacked to plant expression vector pCAMBIA1301. (B) *nifB*, *nifK* and *nifD* stacked to plant expression vector pCAMBIA1301. (C) The recombinant expression vector pCAMBIA1301-*nifBHDK* carrying four *nif* genes (*nifB*, *nifH*, *nifD* and *nifK*).

Then, the four recombinant satellite plasmids (pOSB103-*nifB*, pOSB202-*nifH*, pOSB104-*nifD* and pOSB202-*nifK*) were assembled to plant expression vector pCAMBIA2300 via Cre/loxP recombination, since both satellite vector and pCAMBIA1301 (receptor vector) contain loxP sequences. Recombinant satellite vector and pCAMBIA1301 were co-transformed into *Escherichia coli* SW106 containing the in vivo recombinant enzyme (Cre). Using the Cre/loxP recombination system and the homing endonuclease, which is rare in nature, the four gene expression cassettes were stacked one by one on the plant expression vector pCAMBIA1301 through rounds of in vivo recombination [23]. As shown in Fig 1A, the first round of in vivo Cre/loxP-mediated recombination reactions were performed between pCAMBIA1301 and pOSB103-*nifB*, generating pCAMBIA1301-*nifB*. Then, the pCAMBIA1301-*nifB* was digested with *Pac*I to remove satellite vector framework and redundant loxP. The digested pCAMBIA1301-*nifB* with *Pac*I was used to do the second of round of in vivo Cre/loxP-mediated recombination reactions with pOSB202-*nifH*, generating pCAMBIA1301-*nifBH* which carried both *nifB* and *nifH* expression cassettes. Then, the pCAMBIA1301-*nifBH* was digested with *Asc*I to remove satellite vector framework and redundant loxP.

Similarly, *nifB*, *nifD* and *nifK* were co-integrated on pCAMBIA2300 through several rounds of in vivo Cre/loxP-mediated recombination reactions (Fig 1B). Another first round of in vivo Cre/loxP-mediated recombination reactions was performed between pCAMBIA1301-*nifB* and pOSB202-*nifK*, producing pCAMBIA1301-*nifBK* which was further digested with *Asc*I to remove satellite vector framework and redundant loxP. Then, the second round of in vivo Cre/loxP-mediated recombination reactions was performed between pCAMBIA1301-*nifBK* and pOSB104-*nifD*, generating pCAMBIA1301-*nifBKD* which was further digested with I-*Sce*I to remove satellite vector framework and redundant loxP.

Finally, pCAMBIA1301-*nifBKD* was digested with *Asc*I, generating a large DNA fragment carrying both *nifD* and *nifK* expression cassettes (Fig 1C). The large DNA fragment was ligated to pCAMBIA1301-*nifBH* digested by *Asc*I, producing pCAMBIA1301-*nifBHDK*. The recombinant plasmid pCAMBIA1301-*nifBHDK* carrying four expression cassettes of *nif* genes (*nifB*, *nifH*, *nifD* and *nifK*) was introduced into cotton. As shown in Fig 1C, the expressions of *nifB*, *nifH*, *nifD* and *nifK* were under control of nos, 35S, ocs, and 35S promoters, respectively.

### Generation of transgenic cotton plants expressing *nifBHDK*

**Fig 2:**
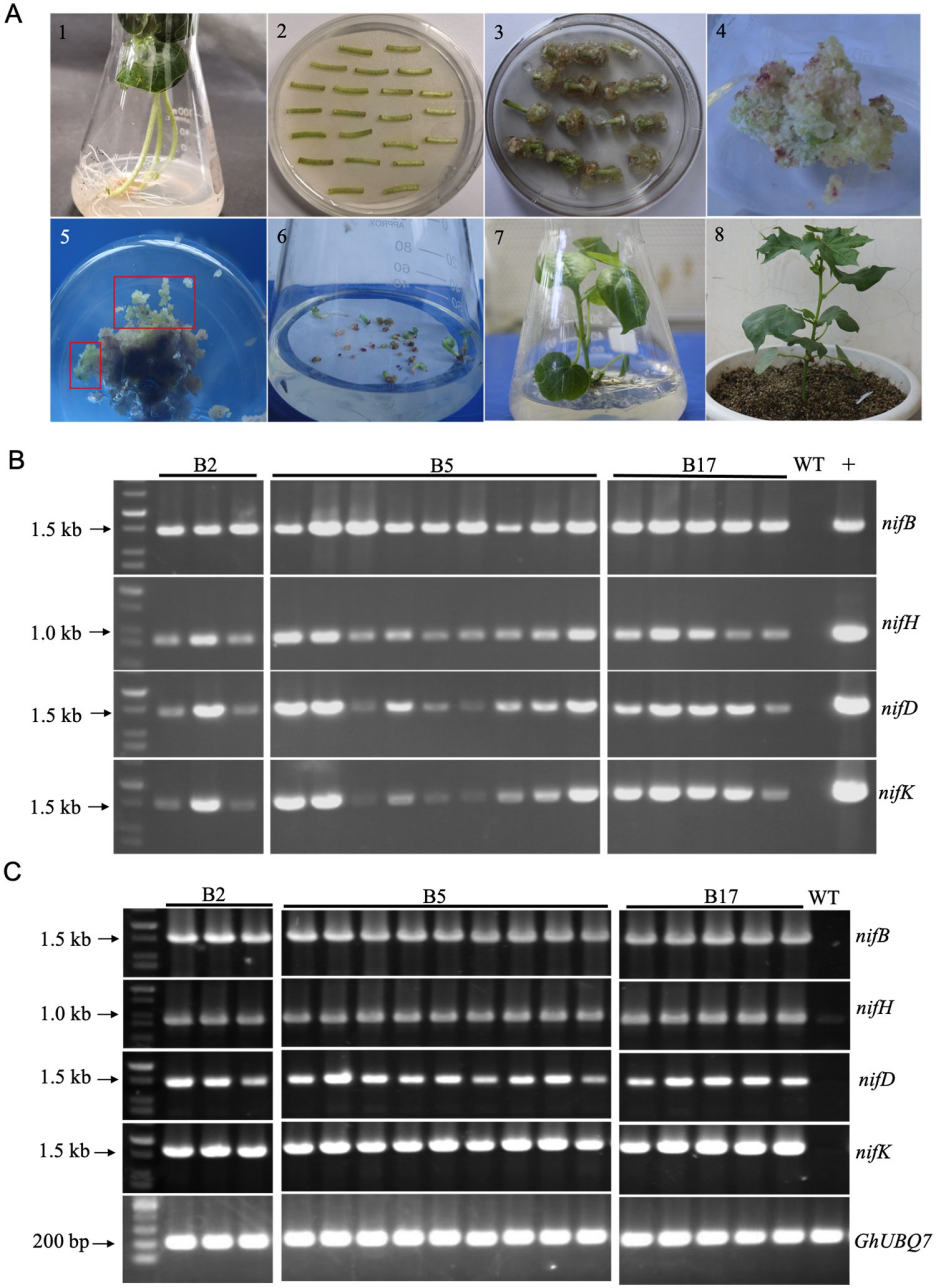
Genetic transformation of cotton plants with four *nif* genes (*nifB*, *nifH*, *nifD* and *nifK*) carried in recombinant expression vector pCAMBIA1301-*nifBHDK*. (A) Schematic diagram of four *nif* genes (*nifB*, *nifH*, *nifD* and *nifK*) under control of different promoters. LB: left border; RB right border. (B) Genetic transformation of cotton plants. 1. Aseptic cotton seedlings. 2. Co-culture of explants with *A*. *tumefaciens*. 3. Induction of resistant callus. 4. Primary callus. 5. Embryogenic callus. 6. Embryoids. 7. Regenerated plants. 8. The grafted transgenic cotton plants of T_0_ generation. (C) PCR analysis of the four *nif* genes (*nifB*, *nifH*, *nifD* and *nifK*) in the three homozygous transgenic cotton lines (B2, B5 and B17) of T_3_ generation. Of the 17 plants analyzed by PCR, 3 plants from cotton line B2, 9 plants from cotton line B5 and 5 plants from cotton line B17. WT: non-transformed plant, +: Expression vector pCAMBIA1301-*nifBHDK* as positive control. (D) RT-PCR analysis of the expression of *nifB*, *nifH*, *nifD* and *nifK* in 17 plants from the three homozygous transgenic cotton lines (B2, B5 and B17) of T_3_ generation. WT: non-transgenic cotton plant R15. GhUBQ7: cotton reference gene.

**Fig 3:**
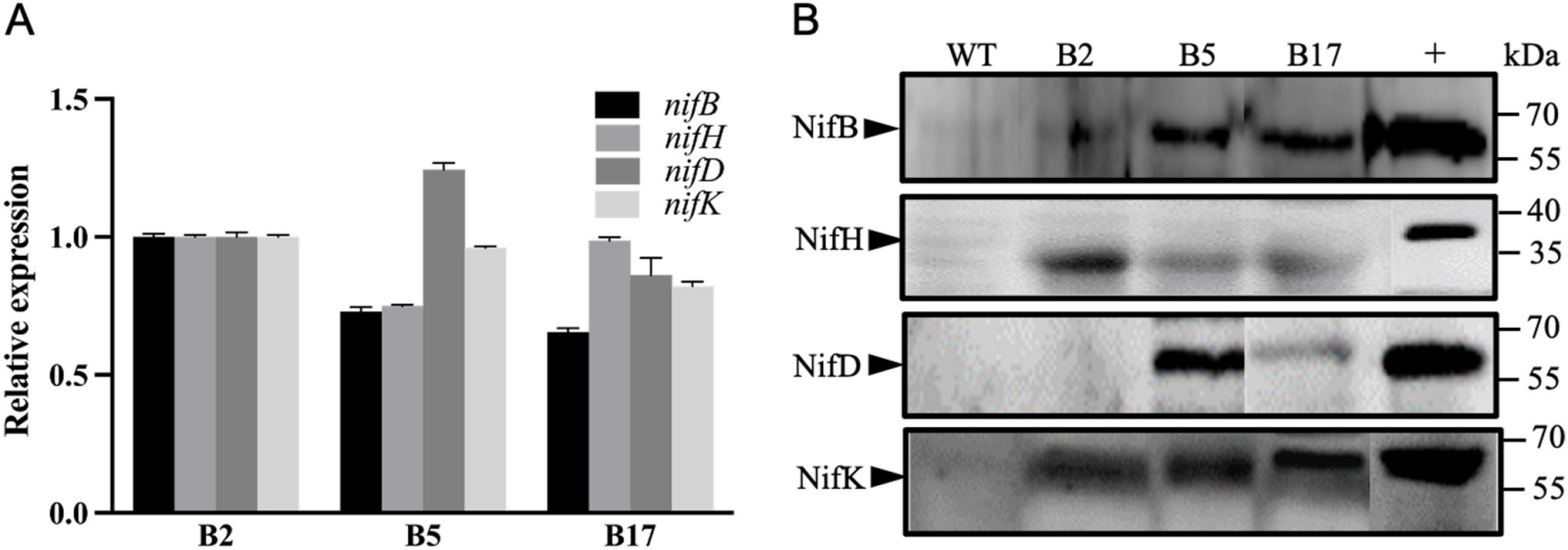
Co-expression of four Nif proteins (NifB, NifH, NifD and NifK) in three homozygous transgenic cotton lines of T_3_ generation. (A) qRT-PCR analysis of expression levels of four *nif* genes in three homozygous cotton lines (B2, B5 and B17). (B) Western blot analysis of expression of four Nif proteins in three homozygous cotton lines (B2, B5, and B17). WT: non-transgenic cotton plant R15. +: N_2_-fixing *P*. *polymyxa*WLY78 as positive control.

The expression vector pCAMBIA1301-*nifBHDK*, was introduced into upland cotton (*Gossypium hirsutum L*.) R15 mediated by *A*. *tumefaciens*. And four *nif* genes (*nifB*. *nifH*, *nifD* and *nifK*) were under control of different promoters (Fig 2A). The cotton seeds whose coats were removed after disinfection treatment were planted in the seedling culture medium and cultured in light incubator (16 h light/8 h dark) at 28°C for 7 days (Fig 2B-1). Then, the hypocotyl was cut into segments with about 1 cm in length, and these segments were co-cultured with solution (OD600 to 0.2–0.4) of *A*. *tumefaciens* carrying the expression vector pCAMBIA1301-*nifBHDK* for 48 h (Fig 2B-2). Afterwards, the cotton hypocotyls were transferred to the resistant callus induction medium for induction. Primary calli were generated at both ends of the incision (Fig 2B-3). The calli were taken from the ends of the hypocotyls, placed on new selection medium, and cultured for another 1–2 months (Fig 2B-4). Primary embryoids were produced by the calli (Fig 2B-5) and embryoid body was produced when being cultured on an embryoid induction medium (Fig 2B-6). The embryoid body was regenerated into cotton seedlings in tissue culture conditions (Fig 2B-7). The lignified transgenic seedlings were grafted onto the cotton rootstock seedlings (25-day old island cotton), and the transgenic cotton of T_0_ generation was continuously cultivated in greenhouse conditions (Fig 2B-8).

T_0_ transgenic cotton plants were screened by PCR to confirm the presence of the *nifB*, *nifH*, *nifD* and *nifK* gene sequences. Of the 25 transgenic cotton plants assayed, 11 lines of transgenic cotton carrying *nifBHDK* genes were obtained, with the positive rate of transformation being 44%. Seeds of T_1_ generation were sown in the experiment fields for 2 consecutive generations. Three transgenic homozygous lines (namely B2, B5 and B17) of T_3_ generation were obtained by PCR screening. Furthermore, PCR analysis of the 17 plants from the three homozygous lines B2, B5 and B17 showed that all of the four foreign genes *nifB*, *nifH*, *nifD* and *nifK* were introduced into these cotton plants (Fig 2C). RT-PCR showed that all of the four genes *nifB*, *nifH*, *nifD* and *nifK* were co-transcribed in all of the 17 transgenic cotton plants (Fig 2D).

### Co-expression of four Nif proteins (NifB, NifH, NifD and NifK) in cottons

qRT-PCR and western blotting analysis were performed to determine whether all of the four *nif* genes (*nifB*, *nifH*, *nifD* and *nifK*) were co-expressed in transgenic cottons. qRT-PCR showed that all of the four *nif* genes (*nifB*, *nifH*, *nifD* and *nifK*) were transcribed in the three transgenic homozygous lines (B2, B5 and B17) of T_3_ generation (Fig 3A). The four *nif* genes (*nifB*, *nifH*, *nifD* and *nifK*) were transcribed in B2 transgenic cottons at similar levels, while *nifD* and *nifK* in B5 transgenic cottons and *nifH*, *nifD* and *nifK* in B17 transgenic cottons were more highly transcribed.

Western blotting analysis with four antibodies against the proteins NifB, NifH, NifD and NifK of *P*. *polymyxa* WLY78 was performed to investigate expression of the four Nif proteins in the three transgenic homozygous lines (B2, B5 and B17) of T_3_ generation. All of NifB, NifH, NifD and NifK proteins were detectable in B5 and B17 transgenic cottons, while only NifB, NifH and NifK were detectable in B2 transgenic cottons (Fig 3B). The molecular weights of NifB, NifD and NifK from transgenic cotton plants were similar with those from *P*. *polymyxa* WLY78, respectively. However, the molecular weight of NifH was smaller than that from *P*. *polymyxa* WLY78. The transgenic cotton plants did not show any phenotypic differences compared to the wild-type cotton plants (S1 and S2 Figs), indicating that production of all NifB, NifH, NifD and NifK did not significantly affect plant metabolism or development.

## Discussion

Biological nitrogen fixation requires a large number of *nif* genes. How to engineer a large number of *nif* gene into eukaryotic organism is still a challenge. In this study, the four genes (*nifB*, *nifH*, *nifD* and *nifK*) from *P*. *Polymyxa* WLY78 were constructed in plant expression vector pCAMBIA1301 by using the Cre/loxP recombination system, yielding the recombinant expression vector pCAMBIA1301-*nifBHDK*. Then, pCAMBIA1301-*nifBHDK* was introduced to cotton. Of the 25 transgenic cotton plants assayed by PCR, 11 lines of transgenic cotton carried *nifBHDK* genes. These results suggest that the Cre/loxP recombination is one of effective approaches to assembly multiple genes in a vector. Our results support that Cre is recognized as the best site-specific recombinase for multiple gene assembly [23, 27–29].

qRT-PCR showed that all of the four genes (*nifB*, *nifH*, *nifD* and *nifK*) were co-transcribed in three homozygous transgenic lines (B2, B5 and B17) of T_3_ generation. The transcription levels of the four *nif* genes exhibited variation among the three homozygous transgenic lines (B2, B5 and B17). The difference of transcription levels of the four *nif* genes may imply that the four *nif* genes were integrated in different positions of transgenic cotton genome. Western blotting analysis showed that NifB, NifH, NifD and NifK were co-produced in B5 and B17 cottons. Whereas, NifB, NifH and NifK were detectable in B2 cottons, but NifD was not. We are not clear what leads to no production of NifD in B2 cottons. The molecular weights of NifB, NifD and NifK from transgenic cottons are identical to those of the original *P*. *polymyxa*WLY78, but the molecular weight of NifH protein is smaller than that of *P*. *polymyxa*WLY78. The data indicate that NifH protein was degraded in cotton plants. In constrast, our recent results have revealed that the NifH expressed in *Saccharomyces cerevisiae* has an identical molecular weight with that of *P*. *polymyxa* NifH [30]. The data indicate that the stability of the expressed Nif proteins in yeast and plant shows variation. It was reported that NifD protein expressed in mitochondria of *S*. *cerevisiae* was smaller than the original *A*. *vinelandii* NifD [31]. Recently, it has revealed that the NifD sequences from *A*. *vinelandii* and *K*. *oxytoca* in position 99 and 100 were cleaved in yeast mitochondria or tobacco (*Nicotiana benthamiana*) mitochondria by yeast or plant mitochondrial protease and the NifD variants whose amino acid residue at 98 or at 100 was substituted by other amino acid residue were resistant to degradation [32, 33]. These results indicate that stability of the expressed Nif proteins in eukaryotes from different N_2_-fixing bacteria shows some variation.

## Conclusion

NifB, NifH, NifD and NifK are four critical proteins in synthesis of nitrogenase. Expression of the *nifB*, *nifH*, *nifD* and *nifK* is essential to generate plants that are able to fix atmospheric N_2._ In this study, the four genes (*nifB*, *nifH*, *nifD* and *nifK*) from *P*. *Polymyxa* WLY78 were assembled in plant expression vector pCAMBIA1301 via Cre/LoxP recombination system. Then, the four *nif* genes carried in the expression vector were co-introduced into upland cotton R15 using *A*. *tumefaciens*-mediated transformation. Homozygous transgenic cotton lines B2, B5 and B17 of T_3_ generation were selected by PCR and RT-PCR. qRT-PCR showed that *nifB*, *nifH*, *nifD* and *nifK* were co-expressed in the transgenic cottons at similar levels. Western blotting analysis demonstrated that NifB, NifH, NifD and NifK were co-produced in the homozygous transgenic cottons. Co-expression of the four critical Nif proteins (NifB, NifH, NifD and NifK) in cottons represents an important step in engineering nitrogenase biosynthetic pathway to non-legume plants.

## Supporting information

S1 Fig Growth of wild-type (WT) cotton and homozygous transgenic lines B2, B5 and B17. (TIF)

S2 Fig Growth of wild-type (WT) cotton and homozygous transgenic lines B2, B5 and B17. (TIF)

S1 Table The codon-optimized sequences of Nif proteins. (PDF)

S2 Table PCR primers for cloning *nif* genes (*nifB*, *nifH*, *nifD* and *nifK*) for construction of expression vector. (PDF)

S3 Table Primers used for PCR and RT-PCR for amplification of *nif* genes in transgenic cotton plants. (PDF)

S4 Table Primers for qRT-PCR analysis of expression of four *nif* genes (*nifB*, *nifH*, *nifD* and *nifK*) in transgenic cotton plants. (PDF)

S1 Raw images (PDF)
