# Correction: Co-expression of nitrogenase proteins in cotton (*Gossypium hirsutum *L.)

**DOI:** 10.1371/journal.pone.0315496

**Published:** 2024-12-05

**Authors:** Yimin Shang, Wenfang Guo, Xiaomeng Liu, Lei Ma, Dehu Liu, Sanfeng Chen, the PLOS ONE Editors

This correction notice is issued to resolve errors with Figs 2 and 3 presented in [1].

The Fig 2A schematic diagram of four *nif* genes under control of different promoters was inadvertently excluded from the published Fig 2A and the published figure was labeled incorrectly. An updated Fig 2 presenting the schematic and correct labelling is provided with this notice.

All panels in Fig 3B panels contain unmarked splice lines. The original blots underlying the published Fig 3B results are provided in the S1 Raw images file available with the published article [1]. Upon review of the article and the published supporting information files, several additional concerns were raised:

- There does not appear to be consistency in the loading order for the different blots reported in the S1 Raw images file of [1].
- In Fig 3B and the corresponding underlying data, the NifH positive control appears to be a different size than the bands for the proteins expressed by cotton lines B2, B5 and B17 (approx. 40kDa vs approx. 35 kDa respectively).

Regarding the inconsistencies in the blot loading order, the authors commented that some blots in the Supporting Information files available with the published article [1] were mislabeled, as evidenced by the loading order reported in their lab books. The authors provided the updated Fig 3 below and an updated S1 Raw images file (S1 File with this notice) that presents the correct loading order. In the updated results, the original finding that NifH is expressed in the B2 transgenic cotton line is no longer supported. Nevertheless, the B5 and B17 results uphold the article’s main conclusion that the four Nif proteins can be co-expressed in transgenic cotton.

Regarding the difference in NifH protein molecular weight, the authors stated that NifH protein in *Paenibacillus sabinae*, used as positive control, is larger than NifH protein in *Paenibacillus polymyxa* from which the transgenic plant lines were derived. In addition, the authors stated that the observed size difference may be due to NifH cleavage.

The authors clarified that the Nif antibodies used in this study were polyclonal antibodies produced by Shenzhen Huada Gene Technology Co., Ltd, China. Data demonstrating the specificity of these antibodies are provided with this notice in S2 File. The secondary antibodies used in the western blot experiments were HRP-Goat Anti-rabbit IgG(H+L) from Huaxingbio Biotechnology, Bejing, China (Catalog: HX2031).

**Fig 2:**
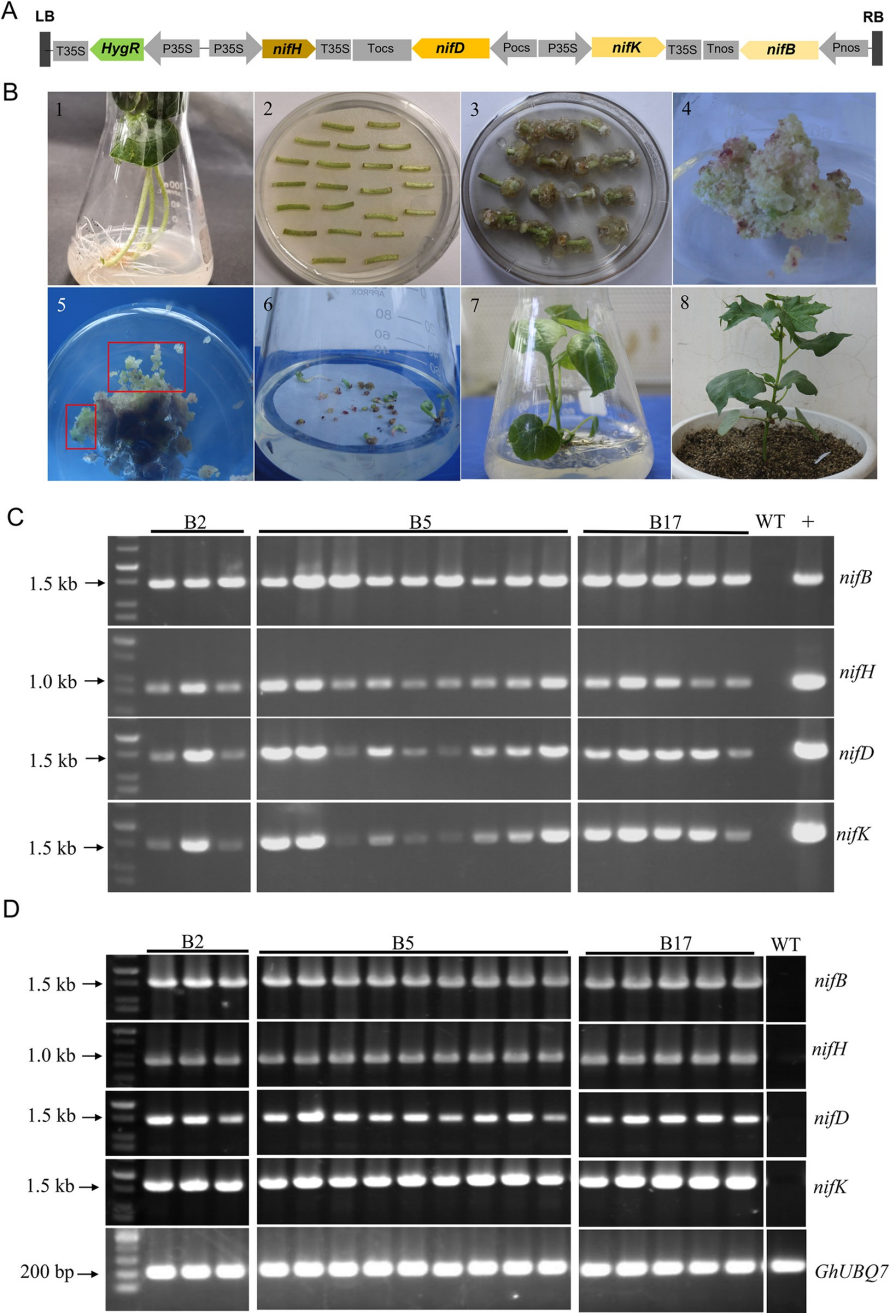
Genetic transformation of cotton plants with four *nif* genes (*nifB*, *nifH*, *nifD* and *nifK*) carried in recombinant expression vector pCAMBIA1301-*nifBHDK*. (A) Schematic diagram of four *nif* genes (*nifB*, *nifH*, *nifD* and *nifK*) under control of different promoters. LB: left border; RB right border. (B) Genetic transformation of cotton plants. 1. Aseptic cotton seedlings. 2. Co-culture of explants with *A*. *tumefaciens*. 3. Induction of resistant callus. 4. Primary callus. 5. Embryogenic callus. 6. Embryoids. 7. Regenerated plants. 8. The grafted transgenic cotton plants of T_0_ generation. (C) PCR analysis of the four *nif* genes (*nifB*, *nifH*, *nifD* and *nifK*) in the three homozygous transgenic cotton lines (B2, B5 and B17) of T_3_ generation. Of the 17 plants analyzed by PCR, 3 plants from cotton line B2, 9 plants from cotton line B5 and 5 plants from cotton line B17. WT: non-transformed plant, +: Expression vector pCAMBIA1301-*nifBHDK* as positive control. (D) RT-PCR analysis of the expression of *nifB*, *nifH*, *nifD* and *nifK* in 17 plants from the three homozygous transgenic cotton lines (B2, B5 and B17) of T_3_ generation. WT: non-transgenic cotton plant R15. GhUBQ7: cotton reference gene.

**Fig 3:**
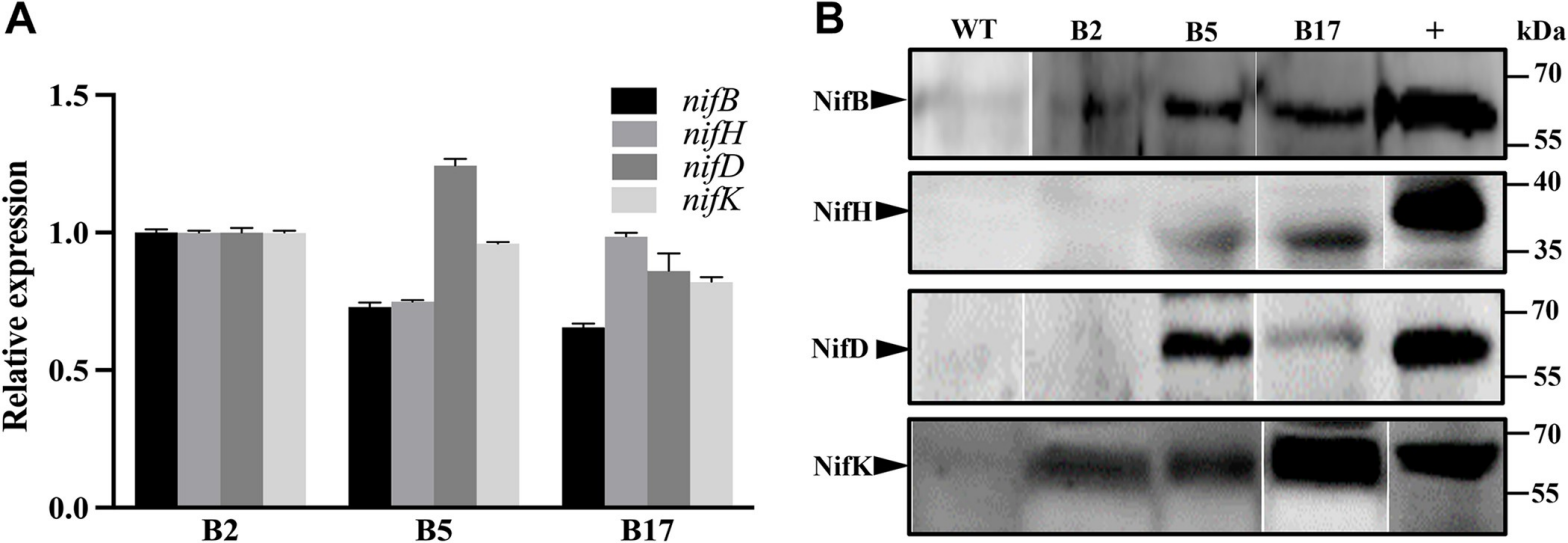
Co-expression of four Nif proteins (NifB, NifH, NifD and NifK) in three homozygous transgenic cotton lines of T_3_ generation. (A) qRT-PCR analysis of expression levels of four *nif* genes in three homozygous cotton lines (B2, B5 and B17). (B) Western blot analysis of expression of four Nif proteins in three homozygous cotton lines (B2, B5, and B17). WT: non-transgenic cotton plant R15. +: N_2_-fixing *P*. *polymyxa*WLY78 as positive control.

## Supporting information

S1 File Updated raw data underlying Fig 3B with corrected labeling. (PDF)

S2 File Antibody validation data. (TIF)
