# Supplementary figures and images for: Co-expression of nitrogenase proteins in cotton (Gossypium hirsutum L.)
Source: PLoS One. 2023 Aug 24;18(8):e0290556. doi: 10.1371/journal.pone.0290556 (PMC10449186; doi:10.1371/journal.pone.0290556)

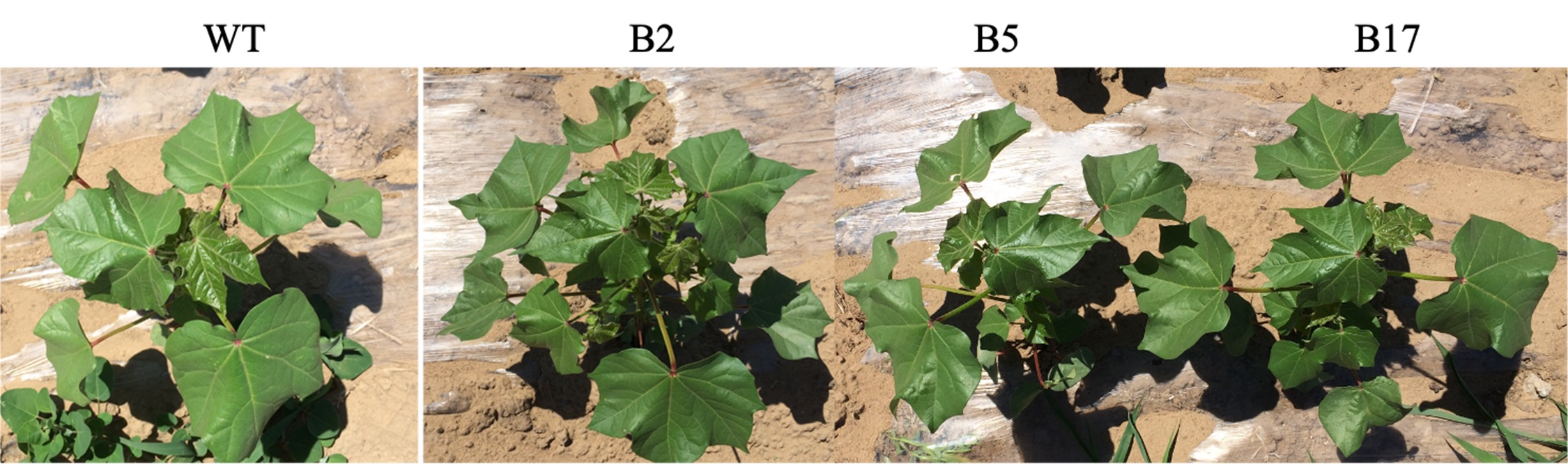

Supplement: S1 Fig — (TIF) [file pone.0290556.s001.tif]

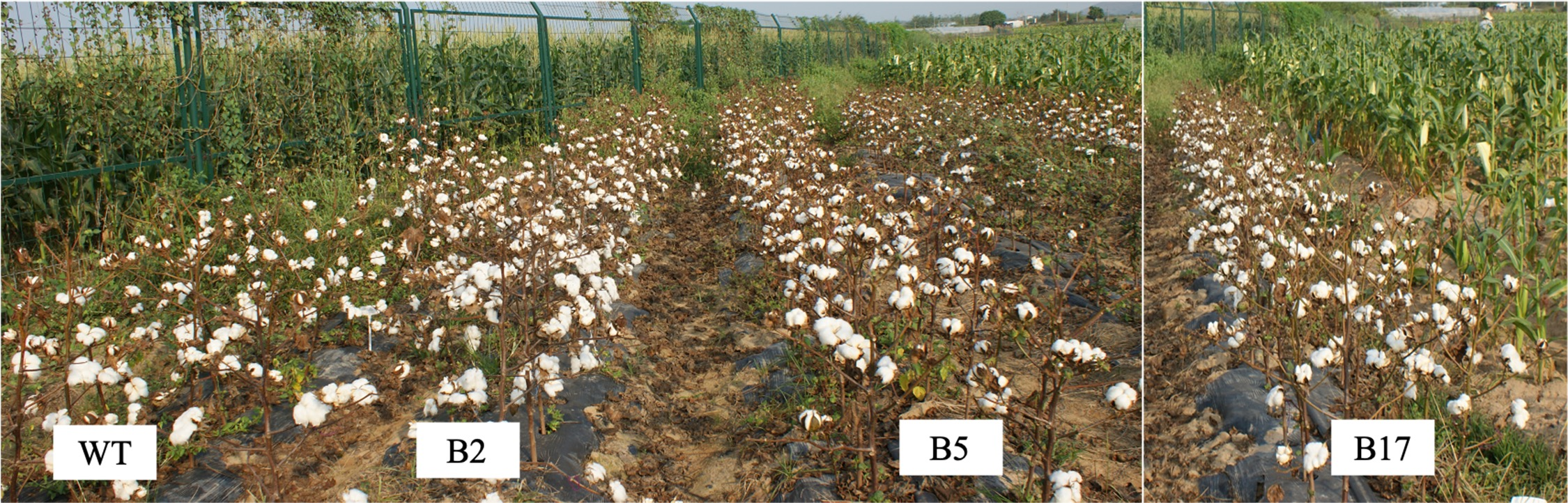

Supplement: S2 Fig — (TIF) [file pone.0290556.s002.tif]
