# Supplementary material for: Co-expression of nitrogenase proteins in cotton (Gossypium hirsutum L.)
Source: PLoS One. 2023 Aug 24;18(8):e0290556. doi: 10.1371/journal.pone.0290556 (PMC10449186; doi:10.1371/journal.pone.0290556)
Supplement: S1 Table — (PDF) [file pone.0290556.s003.pdf]

NifB<br>MDSLADLSETPLALETLRRHPCYNEEAHRYFARIHLPVAPACNIQCHYCNRKFDCVNESRPGVVSELLT<br>PEQAASKTYGVAAQLMQLSVVGIAGPGDPLANAEATFDTFRRVRETVKDVIFCLSTNGLTLIRHIDRIVE<br>LGISHVTITINAVDPVVGSRIYGWVYDEGKRYAGEEAARLLIDRQLAGLKMLASRGVLCKVNSVLIPEV<br>NDAHLPEVARVVKEHGAVLHNIMPLIIAPGSRYEQEGMRAPRPRLVRQLQEQCAEAGAVIMRHCRQCR<br>ADAIGLLGEDRNQDFTWENIAAAPPMDEEARAQFQKELDEKVRVRMERKEGQSHHKQPSTGAGCSCP<br>LSGDKPEASFTSKPVLIAVASRGGGKVNQHFGRAKEFMIYESDGTIVNFIGIRKVQSYCHGKADCNGDK<br>AETIKEILSMVHDCALLLSSGIGEAPKEALQEAGVLPIVCGGDIEESVLEYVKFLRYMYPVQTGKGSKR<br>NKGVKGNHSDLPIEHFGG
NifH<br>MRQIAFYGKGGIGKSTTSQNTLAQLATKFKQKIMIVGCDPKADSTRLILNTKAQQTVLHLAAERGTVE<br>DLELEDVVQKGFGDILNVECGGPEPGVGCAGRGIITAINFLEEEGAYEGLDFVSYDVLGDVVCGGFAM<br>PIREKKAQEIYIVCSGEMMAMYAANNIARGILKYANSGGVRLGGLICNSRNTDLEAELITELARRLNTQ<br>MIHFLPRDNVVQHAELRRMTVTQYNPEHKQAAEYEELAGKILNNDMLTVPTPISMEDLEDLLMEFGII<br>EDEETAINKAEASGQ
NifD<br>MSSIVDKGKQIVEEILEVYPKKAKKDRTKHFEIADEELVNCGTCSIKSNMKSRPGVMTARGCAYAGSK<br>GVVWGPIKDMVHISHGPIGCGQYSWGTRRNYANGILGIDNFTAMQITSNFQEKDIVFGGDKKLEVICRE<br>IKEMFPLAKGISVQSECPVGLIGDDIGAVAKKMTEELGIPVIPVRCEGFRGVSQSLGHHIANDAIRDFLM<br>GRRELKECGPYDVSIIGDYNIGGDAWASRILLEEMGLRVIAQWSGDGTINELGIAHKSKLNLIHCHRSM<br>NYMCTTMEQEYGIPWMEYNFFGPTKTMESLRAIAARFDETIQEKCEQVIAQYMPQMEAVIRKYRPRLE<br>GKKVMLLIGGLRARHTIGAYEDLGMEIVATGYEFAHKDDYEKTFPDVKEGTILYDDPTAYELEELAQRL<br>NIDLMGAGVKEKYVYHKMGIPFRQMHSWDYSGPYHGFDGFKIFARDMDMTINSPVWSLLPSRQTAEV<br>PV
NifK<br>MEPAALTADCGGAGMSERPNIVDHNQLFRQDKYVRQREEKRAFEAPCSPEEVTDTLEYTKTKEYKDK<br>NFARTAVVVNPAKACQPLGAVMAALGFEKTLPFIHGSQGCTAYFRSHLARHFKEPVPAVSTSMTEDAAV<br>FGGMRNLIDGIENCIALYQPEMIAVCTTCMAEVIGDDLSAFLANARQEGVLPEDMPVPFANTPSFSGSHI<br>TGYDAMLRSVLETLYNKSGRTAQPGHELKLNVLLGFDGYTGNFAEMRRMLGMFGATYTILGDHSSNF<br>DSGATGEYSYYYGGTPLEDVPKAADAAGTLAIQQYSLRKTLGYMKQTWGQQVSSISTPLGIRATDRLL<br>EEISRLSGREIPEALKQERARIVDAMMDSHAYLHGKRVAMAGDPDMLIGLIGFCLELGMEPVHIVCSNG<br>DRKFEKEAELLLKSSPYGAEATVHSGQDLWHMRSLLFQDPVDLAIGSSHLKFAAKEAEIPLLRVGFPIFD<br>RHHLHRYPIIGY
