# Supplementary material for: Co-expression of nitrogenase proteins in cotton (Gossypium hirsutum L.)
Source: PLoS One. 2023 Aug 24;18(8):e0290556. doi: 10.1371/journal.pone.0290556 (PMC10449186; doi:10.1371/journal.pone.0290556)
Supplement: S2 Table — (PDF) [file pone.0290556.s004.pdf]

Primer name	Sequences（5'-3'）	Target
FnifB	CCGCTCGAGATGGACTCTTTGGCTGATTTGTC<br>*Xho* I	*nifB* gene
RnifB	GGCCTGTACATTAACCTCCAAAATGTTCAATTGGC<br>*Bsr*G I	
FnifH	CCGCTCGAGATGAGACAAATTGCTTTTTACGG<br>*Xho* I	*nifH* gene
RnifH	GGCCTGTACATTACTGACCGGAAGCCTCAGGAAAT<br>*Bsr*G I	
FnifD	CCGCTCGAGATGTCTAGTATTGTTGATAAGGGT<br>*Xho* I	*nifD* gene
RnifD	GGCCTGTACATTAAACTGGAACCTCAGCAGTCTG<br>*Bsr*G I	
FnifK	CCGCTCGAGATGGAGCCTGCTGCCTTGACTGC<br>*Xho* I	*nifK* gene
RnifK	GGCCTGTACATTATCTAACCAAATCAAAAGAATG<br>*Bsr*G I	
