# Supplementary material for: Co-expression of nitrogenase proteins in cotton (Gossypium hirsutum L.)
Source: PLoS One. 2023 Aug 24;18(8):e0290556. doi: 10.1371/journal.pone.0290556 (PMC10449186; doi:10.1371/journal.pone.0290556)
Supplement: S3 Table — (PDF) [file pone.0290556.s005.pdf]

Primer name	Sequences（5’-3’）	Purpose	Size
nifB-F	GCTGATTTGTCTGAAACCCCA	*nifB*	1425 bp
nifB-R	ACTTCCCTTACCAGTCTGAACA		
nifH-F	GGTAAGGGTGGTATCGGTAAATC	*nifH*	830 bp
nifH-R	TCAGCTTTGTTAATTGCGGTTTC		
nifD-F	GGGTAAGCAGATCGTTGAGG	*nifD*	1414 bp
nifD-R	AGCAGTCTGTCTTGATGGCA		
nifK-F	GGAGGTGCCGGTATGTCTG	*nifK*	1509 bp
nifK-R	CTGCTCCTCCATGACATCCA		
GhUBQ7-F	GAAGGCATTCCACCTGACCAAC	cotton reference gene *GhUBQ7*	198 bp
GhUBQ7-R	CTTGACCTTCTTCTTCTTGTGCTTG		
