# Supplementary material for: Co-expression of nitrogenase proteins in cotton (Gossypium hirsutum L.)
Source: PLoS One. 2023 Aug 24;18(8):e0290556. doi: 10.1371/journal.pone.0290556 (PMC10449186; doi:10.1371/journal.pone.0290556)
Supplement: S4 Table — (PDF) [file pone.0290556.s006.pdf]

Primer name	Sequences（5’-3’）	Purpose	Size
qnifH-F	CAGGAAGAGGTATCATCACAGCCATT	*nifB*	187 bp
qnifH-R	GCAGCGTACATAGCCATCATCTCA		
qnifD-F	GCCGTTGCCAAGAAGATGACAGA	*nifH*	200 bp
qnifD-R	TCACCACCGATATTGTAGTCTCCGATA		
qnifK-F	GTTCCTGCCGTCTCCACCTCTAT	*nifD*	154 bp
qnifK-R	AATCATCACCGATAACCTCTGCCATAC		
qnifB-F	GGTATGAGAGCACCAAGACCAAGATT	*nifK*	171 bp
qnifB-R	TGGAGGAGCAGCAGCAATGTTC		
GhUBQ7-F	GAAGGCATTCCACCTGACCAAC	cotton reference gene *GhUBQ7*	198 bp
GhUBQ7-R	CTTGACCTTCTTCTTCTTGTGCTTG		
