# Supplementary material for: Correction: Co-expression of nitrogenase proteins in cotton (Gossypium hirsutum L.)
Source: PLoS One. 2024 Dec 5;19(12):e0315496. doi: 10.1371/journal.pone.0315496 (PMC11620603; doi:10.1371/journal.pone.0315496)
Supplement: S1 File — (PDF) [file pone.0315496.s001.pdf]

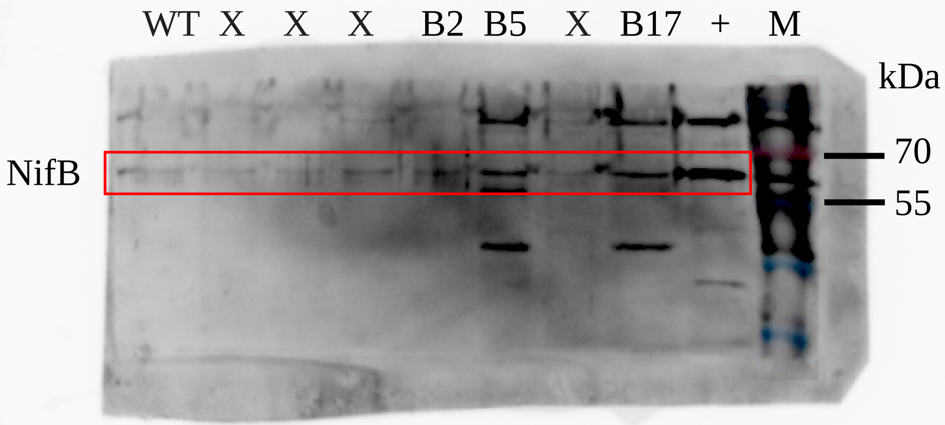

The Western blot original images of NifB in Figure 3B. B2, B5, and B17: transgenic cotton homozygous lines, WT: non-transgenic cotton plant R15. X: the lane not included in the final figure. +: N_2-fixing *P. polymyxa* WLY78 as positive control. M: Protein Marker.

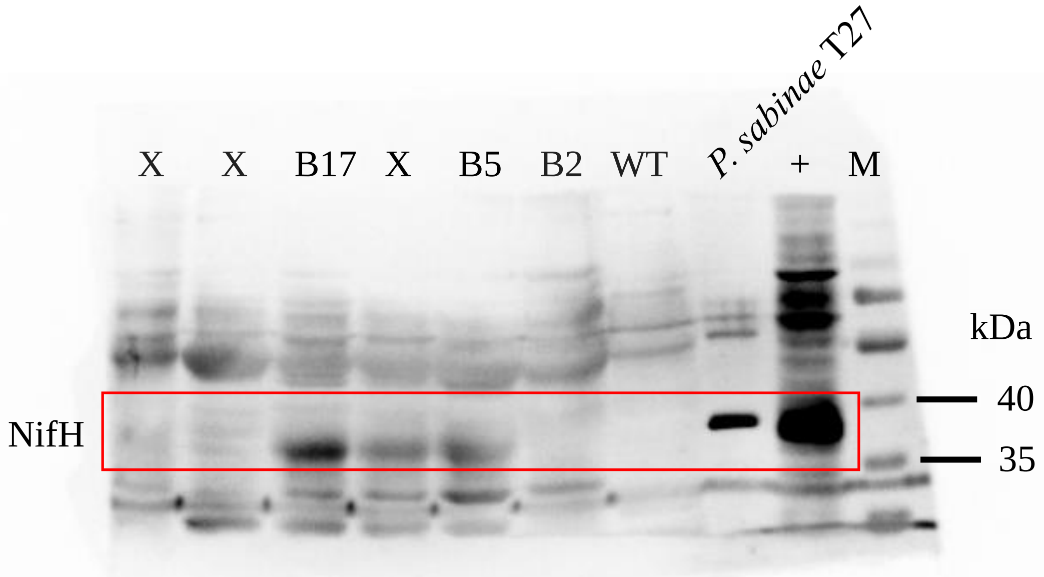

The Western blot original images of NifH in Figure 3B. B2, B5, and B17: transgenic cotton homozygous lines, WT: non-transgenic cotton plant R15. X: the lane not included in the final figure. +: N_2-fixing *P. polymyxa* WLY78 as positive control. M: Protein Marker.

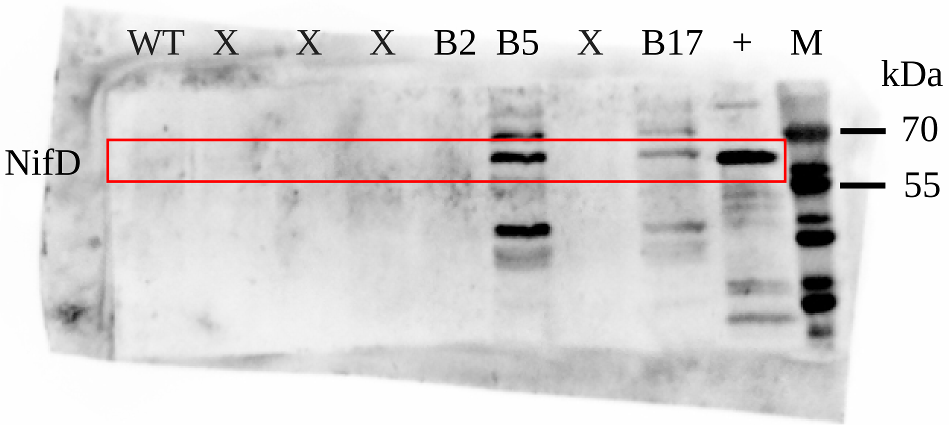

The Western blot original images of NifD in Figure 3B. B2, B5, and B17: transgenic cotton homozygous lines, WT: non-transgenic cotton plant R15. X: the lane not included in the final figure. +: N_2-fixing *P. polymyxa* WLY78 as positive control. M: Protein Marker.

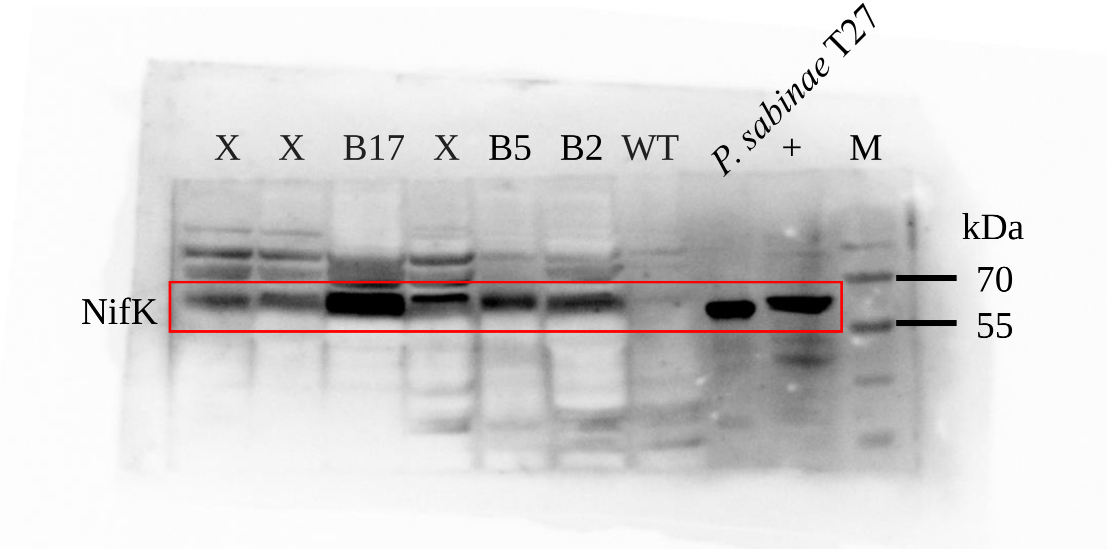

The Western blot original images of NifK in Figure 3B. B2, B5, and B17: transgenic cotton homozygous lines, WT: non-transgenic cotton plant R15. X: the lane not included in the final figure. +: N_2-fixing *P. polymyxa* WLY78 as positive control. M: Protein Marker.
