# Supplementary figures and images for: Correction: Co-expression of nitrogenase proteins in cotton (Gossypium hirsutum L.)
Source: PLoS One. 2024 Dec 5;19(12):e0315496. doi: 10.1371/journal.pone.0315496 (PMC11620603; doi:10.1371/journal.pone.0315496)

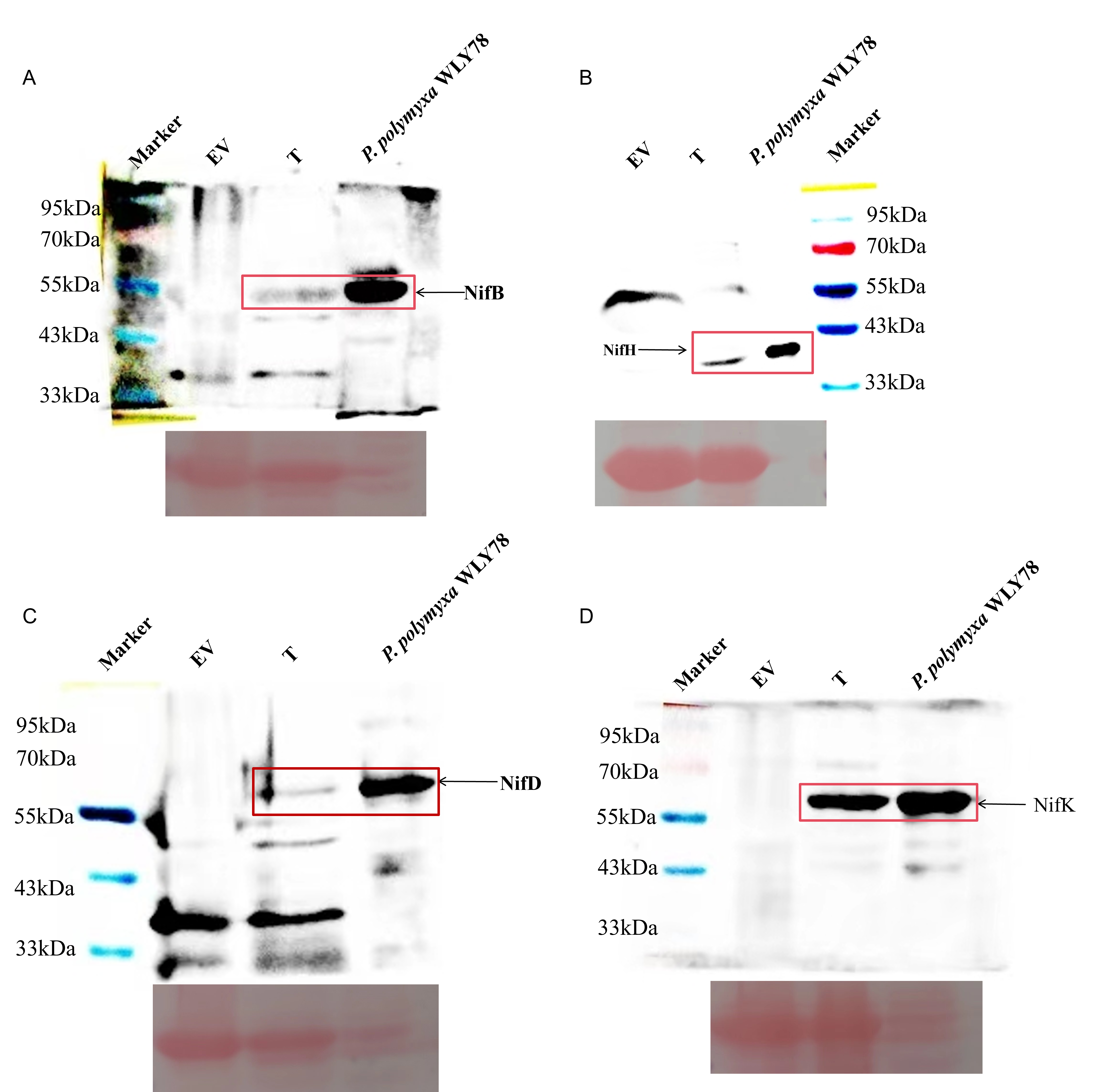

Supplement: S2 File — (TIF) [file pone.0315496.s002.tif]
